# A semantic-based workflow for biomedical literature annotation

**DOI:** 10.1093/database/bax088

**Published:** 2017-11-15

**Authors:** Pedro Sernadela, José Luís Oliveira

**Affiliations:** 1 University of Aveiro, DETI/IEETA, University of Aveiro, Campus Universitário de Santiago, 3810-193 Aveiro, Portugal

## Abstract

Computational annotation of textual information has taken on an important role in knowledge extraction from the biomedical literature, since most of the relevant information from scientific findings is still maintained in text format. In this endeavour, annotation tools can assist in the identification of biomedical concepts and their relationships, providing faster reading and curation processes, with reduced costs. However, the separate usage of distinct annotation systems results in highly heterogeneous data, as it is difficult to efficiently combine and exchange this valuable asset. Moreover, despite the existence of several annotation formats, there is no unified way to integrate miscellaneous annotation outcomes into a reusable, sharable and searchable structure. Taking up this challenge, we present a modular architecture for textual information integration using semantic web features and services. The solution described allows the migration of curation data into a common model, providing a suitable transition process in which multiple annotation data can be integrated and enriched, with the possibility of being shared, compared and reused across semantic knowledge bases.

## Introduction

The continuous growth of scientific literature repositories demands the exploration of automated information extraction tools to access relevant information contained in millions of textual documents and to support translational research (1). In the biomedical domain, progress has been outstanding (2), producing reliable text-mining tools and innovative text-processing algorithms. The combination of these techniques has been increasingly applied to assist bio-curators, allowing the extraction of biomedical concepts such as genes, proteins, chemical compounds or diseases, and thus reducing curation times and cost (3).

Usually, state-of-the-art solutions for biomedical information extraction follow a combination of pre-defined and sequential processes. Natural Language Processing (NLP) techniques (4) are commonly applied as pre-processing tasks to split documents’ text into meaningful components, such as sentences and tokens, assign grammatical categories (a process named part-of-speech tagging), or even apply linguistic parsing to identify the structure of each sentence. Next, concept recognition methods are employed, which involve Named Entity Recognition (NER) (5) to detect the concept mentions, and normalization or disambiguation processes (6) to attribute unique identifiers to each detected entity name. More complete biomedical text-mining solutions also apply relation-mining techniques to identify the events and entity relations that make up complex biological networks. Conventional solutions are focused on investigating and extracting direct associations between two concepts (e.g. genes, proteins, drugs, etc.) (7). The study of these associations has generated much interest, especially in relation to protein–protein interactions (8), drug–drug interactions (9) and relations between chemicals and target genes (10). Recently, interactive text-mining solutions have attracted more attention due to the added benefits of including automatically extracted information in the manual curation processes. With these solutions, the curation time is improved and possible mistakes from computational information extraction results are minimized. Brat (11), MyMiner (12), Argo (13) and Egas (14) are state-of-the-art interactive solutions, aiming to simplify the annotation process.

Nonetheless, these efforts are still hindered by a lack of standardised ways to process the vast amount of data generated (15). This concern can be split in two major challenges. First, there are interoperability issues between information extraction components for concept recognition and relation extraction methodologies. Second, there is no unified way to access the mined information by large-scale applications. Typically, different data models are adopted, hindering a simplified access mechanism and integration with external knowledge bases.

In this manuscript, we propose a modular architecture aiming to support the integration of text-mined information from independent systems. The pipeline developed provides interoperable interfaces for the integration of miscellaneous annotated data, enabling the full exploitation of curated knowledge according to World Wide Web Consortium (W3C) standards. An evaluation study is presented regarding Duchenne Muscular Dystrophy (DMD) disease dataset, showing the integration of two distinct text-mined results into a reusable and searchable knowledge base (available at http://bioinformatics.ua.pt/dmd/scaleus/).

## Background

In recent years, several annotation formats have been advanced to store and distribute biomedical information extraction outcomes. Commonly called annotations, they are generated following a specific structure or format dependent on the extraction system, and integration with external databases and systems is challenging. IeXML (16) was one of the first XML-based implementations to define an exchange format considering annotations and enrichment of text. More recently, the BioC (17) has emerged as a community-supported format for encoding and sharing textual annotations. This simplified approach streamlines data reuse and sharing methods, achieving interoperability for the different text processing tasks by defining connectors to read and write XML annotations. Although they are created to enable interoperability and reusability between text-mined systems, these data structures are not designed to support data exploration and sustainability. To address this issue, it is necessary to develop new research methods to allow fast exploration and distribution of this valuable information.

Emerging semantic web standards and concepts are playing an important role in solving data distribution problems. In the scientific community, this is currently seen as the standard paradigm for data integration and distribution on a web-scale, focused on the semantics and the context of data (18). It allows the construction of rich networks of linked data, offering advanced possibilities to retrieve and discover knowledge (e.g. reasoning). With the increasing adoption of this paradigm to tackle traditional data issues such as heterogeneity, distribution and interoperability, novel knowledge-based databases and systems have been built to explore the potential behind this technology. Essentially, they facilitate the deployment of well-structured data and deliver information in a usable structure for further analyses and reuse. In this way, approaches that combine the benefits of information extraction methods with these semantic systems represent a growing trend, allowing the establishment of curated databases with improved availability (19). Coulet *et al.* (20) provide an overview of such solutions, and describe a use case regarding the integration of heterogeneous text-mined pharmacogenomics relationships on the semantic web. Another case study is described by Mendes *et al.* (21), presenting a translation method for automated annotation of text documents to the DBpedia Knowledge Base (22). A different approach is proposed through the PubAnnotation (23) prototype repository. The notion was to construct a sharable store, where several corpora and annotations can be stored together and queried through SPARQL (24). In this perspective, there is a clear trend to combine text-mined information with semantic web technologies, resulting in improved knowledge exchange and representation. Taking into account these approaches, there is a clear tendency towards workflow construction systems for annotation distribution. However, limitations in the development processes and the existence of software dependencies in the source platforms (25) represent a barrier to adapting and reusing existing solutions for the distribution of distinct annotation structures and formats. The great heterogeneity of biomedical annotations makes it challenging to aggregate results obtained from different tools and systems, with innovative solutions being necessary for multiple annotations’ combination and distribution.

## Materials and methods

**Figure 1.**
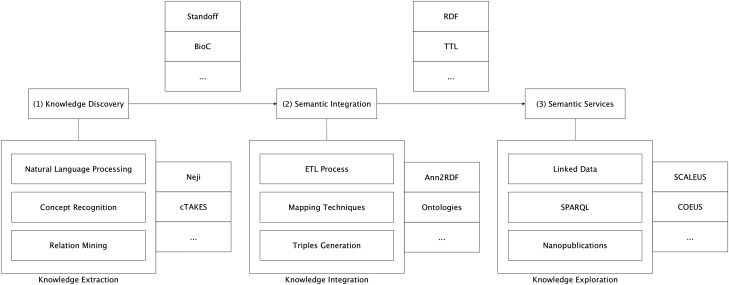
Semantic-based architecture for scientific information integration.

In the background section, we have discussed several alternative methodologies to represent text-mining annotations. Although major contributions have been made in this area, it is still challenging to adapt and link the output of these distinct tools. To address this issue, we implemented a modular architecture able to support the integration of annotations from multiple extraction tools into the semantic web ecosystem (Figure 1).

The proposed approach aims to provide a seamless transition from unstructured information to the semantic web level. The overall architecture is based on a modular and pipelined approach, divided into three interconnected, though independent, components: (i) knowledge discovery; (ii) semantic integration; and (iii) semantic services.

### Knowledge discovery

In this component, textual documents are examined using state-of-the-art text-mining methods for the identification of relevant concepts, respective attributes and relationships. These extraction techniques can be applied by one or by a combination of automated text-mining tools. This means that the architecture does not rely on a single text-mining solution to perform information extraction, with it being possible to aggregate results from several systems. However, each text-mining solution must be delivered as a RESTful Web service to be compliant with the implemented architecture. The deployment of those resources through REST (Representational state transfer) services allows us to standardize how HTTP requests can be performed within the architecture. Service invocations are made through HTTP POST requests, accepting *text/plain* as content type. This simplifies communication between the components developed and facilitates the configuration process for additional text-mining tools and systems integration. The implemented architecture supports NER systems, complete Concept Recognition systems and Relation Extraction systems. In the Results section, a setting with two distinct text-mining solutions is assessed, dealing with different formats and results.

### Semantic integration

Information extraction tools produce several annotation formats. The migration of this data into semantic web format and services provides additional value regarding the share of knowledge. To allow this transition, our methodology is based on *Ann2RDF* modular algorithms (26).

*Ann2RDF* (http://bioinformatics-ua.github.io/ann2rdf/) is based on the creation of modular integration algorithms to deal with the different formats resulting from text-mining tools. The ability to acquire data from several and miscellaneous annotation formats benefits developers, allowing each one to implement and integrate their format in a common interface. Developed algorithms are based on Object Relation Mapping techniques for mapping different data structures to a single representation and on advanced Extract-Transform-and-Load (ETL) procedures to select and extract annotations content based on regular expressions and data parsers such as XPath (XML Path Language). Currently, the system supports the integration of most BioNLP Workshop’s (http://bionlp.org) formats out-of-the-box such as the BioC and Standoff formats, with it also being possible to additionally customize new formats.

After this selection and extraction processes, annotations objects are semantically enriched by using ontology mapping procedures: the system makes use of an external JSON-based configuration file to assist the ontology mapping process. In this configuration file, the mappings between classified concept categories and relation properties (i.e. associations between concepts) are defined to the respective ontology terms. This allows standardization of annotations’ content, e.g. ‘A relatedWith B “to “A dc: relation B’, using for instance, the Dublin Core Ontology (27). Next, there is the possibility to normalize the detected concepts. Due to the existence of many NER tools that do not include concept normalization tasks, the system offers an optional normalization service. The invocation is also performed in the same configuration file, declaring external HTTP POST requests. For this invocation, two properties are needed: the service location and the regular expression to apply to select the desired output. With this external support, services such as BioPortal Annotator (28) (e.g. service: ‘http://data.bioontology.org/annotator?apikey = XXXX’, query: ‘[*].annotatedClass.@id’) or BeCAS (29) (e.g. service: ‘http://bioinformatics.ua.pt/becas/api/text/annotate’, query: ‘*.*.refs’) can be easily integrated, providing an enhanced incorporation of the annotated data and improved simplification for the semantic integration process.

Finally, harmonization methods are responsible for performing an adequate linkage between extracted content and the respective structured model.

To represent the processed data, our architecture model is based on Annotation Ontology (AO) (30), an open representation model for representing interoperable annotations in RDF (Resource Description Framework) which is currently being used by the W3C community (https://www.w3.org/TR/annotation-vocab/). It provides a robust set of methods for connecting web resources, for instance, textual information in scientific publications, to ontological elements, with full representation of annotation provenance, a contextual metadata describing the origin or source (31, 32). By linking new scientific content to computationally defined terms and entity descriptors, AO helps to establish semantic interoperability across the biomedical field. Through this model, existing domain ontologies and vocabularies can be used, creating extremely rich stores of metadata on web resources.

#### Concept model

**Figure 2.**
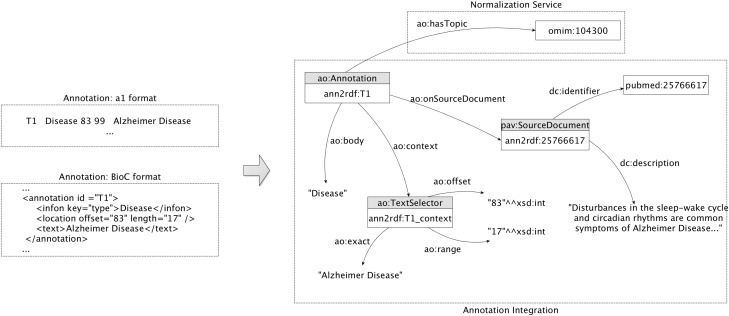
Annotation model: sample extraction of the integration and representation of an annotation related to the ‘Alzheimer disease’.

We reuse the AO core ontology components to describe generated annotations. In Figure 2, we present the adopted core model, using a sample annotation regarding identification of the Alzheimer disease. The central point of the representation includes the URI (e.g. ann2rdf: *T1*), the document source (e.g. Pubmed ID *25766617*), and the respective annotated data (e.g. *Alzheimer Disease*). The text selectors are used to identify the string detected on the document: the *ao: exact* data property represents the linear sequence of characters, i.e. the subject of the annotation, the *ao: offset* data property indicates the distance from the beginning of the document up to a given element or position, and the *ao: range* data property represents the number of characters starting from the offset. Information about the annotation itself is connected through two different properties: the *ao: body* representing the annotated resource and the *ao: hasTopic* indicating the semantic identifier of the detected resource (e.g. OMIM ID *104300*). The identifier is attributed by the normalization service to represent ‘Alzheimer Disease’ annotation due to the inexistence of such information on the previously annotated data. If the annotation data already contemplate a semantic identifier, it is extracted and connected to the annotation graph. Moreover, the annotations are linked to the respective document source through the object property *ao: onSourceDocument* providing a provenance interchange mechanism. By using this simplified model, entity annotations can be easily mapped to a semantic web-compliant format.

#### Relation model

Researchers typically refer to relation extraction as the task of identifying binary relations between concepts (i.e. co-occurrence), and to event extraction as the identification of more complex relationships, involving verbs or normalized verbs (i.e. trigger) to characterize the event type. Event extraction techniques started to become more familiar with the introduction of BioNLP shared tasks (33), allowing the construction of complex conceptual networks.

**Figure 3.**
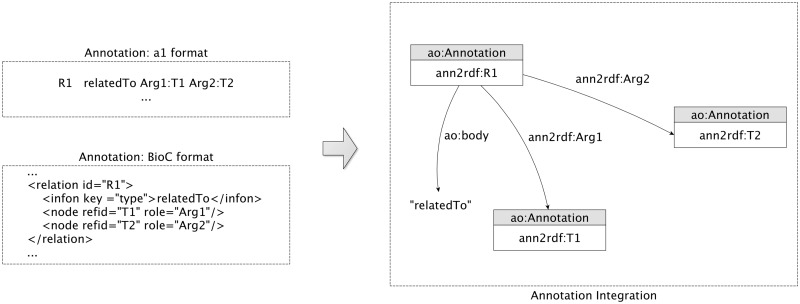
Relation model: sample extraction of the integration and representation of a *relatedTo* annotation relationship.

We introduced new relationships to allow the representation of annotation interactions. To represent the relations (Figure 3), our model essentially connects the binary entities through one additional annotation. The relation is not directly established between the two entities involved due to the possible existence of different specificity in the object property linkage between relations. For this reason, a new annotation is created to associate the two annotations and a respective descriptive relation type is attributed through the *ao: body* property. Regarding the representation of events, our model achieves a similar structure of the relation annotations but with some adjustments, i.e. instead of only representing the binary relation it can represent multiple associations between annotations. Using the representations described, the outcomes of text-mining tools can be easily integrated into a unified model providing semantic web interoperability features for the mined resources.

### Semantic services

The semantic web has gained an increasing role as a suitable environment to solve knowledge representation and interoperability problems, creating accessible and shareable information across application and database boundaries. Its adoption by the life science community allows better standards and technologies to be delivered, making the interconnection across knowledge domains possible and effective. Taking those benefits into account, our flexible solution enables the deployment of several semantic-based systems and services. Developed to support the current need of semantic-web services (18), existing systems explore the potential behind semantic web technology, enabling the quick creation of new knowledge bases for further exploration. COEUS (34), SCALEUS (35) and SADI (36) are some examples of these systems, which can be used along our modular solution.

However, this study is only focused in the implementation and exploration of services residing in the SCALEUS web system. With this adoption, we take advantage of several services, including a database management system with simplified APIs, a SPARQL query engine supporting real-time inference mechanisms, and optimized text searches over the knowledge base. Inference on the Semantic Web is one of the most useful tools to enhance data integration quality, automatically analyzing the content of the data and discovering new relationships. In the deployed system, the SPARQL query engine plus user-defined rules makes it possible to generate new relationships from existing triples, and therefore increase reasoning capabilities by inferring or discovering additional facts about the stored data. Regarding the text-search feature, it offers the ability to perform free-text searches within SPARQL queries. By using this extension, literals are tagged and indexed by an Apache Lucene (http://lucene.apache.org) engine. Essentially, the text index is used to provide a reverse index mapping query strings to URIs. The support of SPARQL Federated Query (https://www.w3.org/TR/sparql11-federated-query/) is also an available feature allowing the execution of distributed queries over different SPARQL endpoints. In this way, the deployment of these semantic services with the combination of existing life science knowledge bases such as the Bio2RDF (37) or the EMBL-EBI RDF Platform (38) provides a well-structured network, in which federated inquiring mechanisms can be easily applied (39, 40).

## Results

The developed architecture, involves a diverse combination of systems and technologies, lying in the intersection of knowledge discovery and semantic web methods. Due to its modularity, several components can be used, providing greater freedom for end-users and offering distinct possibilities for information integration and access.

Regarding the contribution, this study is focused on the implementation of a modular semantic-web workflow for the integration and reuse of multiple text-mined results. To allow this, three main components where developed: (i) Development of literature extraction methods based on RESTfull APIs; (ii) Improvement and adaptation of *Ann2RDF* algorithms for annotations integration and enrichment. (iii) Development and deployment and of a SCALEUS instance, for annotations exploration (available at http://bioinformatics.ua.pt/dmd/scaleus/). In the next sections, we explore and evaluate these components towards a unified workflow for data integration and distribution.

### Information extraction

To demonstrate the feasibility of the implemented solution, we explored a combination of two distinct text-mining solutions. The first solution is Neji (41), a modular framework for biomedical NLP. This open-source framework allows the integration in a single pipeline, as dynamic plugins, of several state-of-the-art methods for biomedical NLP, such as sentence splitting, tokenization, lemmatization, part-of-speech, chunking and dependency parsing. The concept recognition tasks can be performed using dictionary matching and machine learning techniques with normalization. This framework implements a very flexible and efficient concept tree, where the recognized concepts are stored, supporting nested and intersected concepts with one or more identifiers. The architecture of Neji allows users to configure the processing of documents according to their specific objectives and goals, providing very rich and complete information about concepts.

The second tool used in this example is cTAKES (42), an open-source NLP system for information extraction from free text of electronic medical records. The system was designed to semantically extract information to support heterogeneous clinical research. It consists of a sequence of modular components (including sentence boundary detector, tokenizer, normalizer, part-of-speech tagger, shallow parser and NER) that process clinical free-text, contributing to a cumulative annotation dataset. cTAKES was already optimized to explore the characteristics of clinical narratives. By exploring both tools, we expect to maximize coverage in the biomedical and healthcare fields.

Neji and cTAKES services were both deployed with end-user Web interfaces and REST APIs, simplifying the test and validation of our architecture. The dictionaries used in both solutions were retrieved from the 2014 UMLS Metathesaurus database (43), which contains key terminology, classification and coding standards assigned to terms. Each term has a concept unique identifier, to be assigned to each identified concept. Both solutions can perform concept recognition through REST services. In addition, the cTAKES annotator can execute relation extraction techniques between identified concepts. These binary relations are recognized using a rule-based and machine learning components, making it possible to detect interactions such as *degree of* (e.g. degree of pain) or *location of* (e.g. location of pain).

### Evaluation

**Figure 4.**
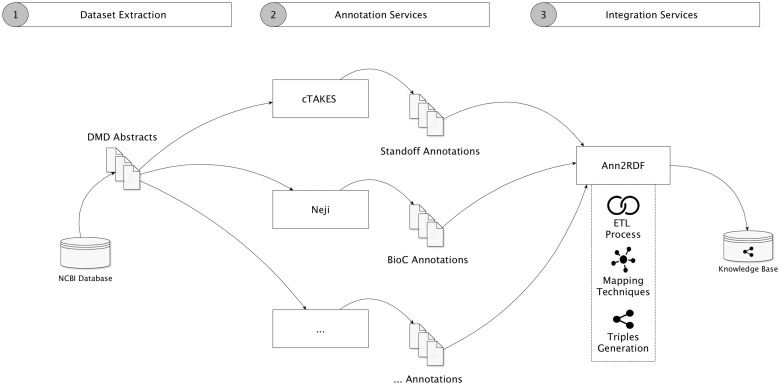
Validation workflow overview. (1) Dataset is extracted from the NCBI database. (2) Neji and cTAKES API services were used for information extraction, generating diverse outputs and formats. Additional annotation services can be used. (3) Annotations are forwarded and integrated into a unified model and stored in an accessible knowledge base.

To validate our architecture, we conducted a case study aimed to create a semantic repository from a dataset related to *DMD*, a rare disease condition affecting 1 in 5000 males at birth. For this case study, we collected a dataset containing 2783 DMD-related abstracts, obtained by accessing the Entrez Programming Utilities interfaces in the NCBI database. Figure 4 shows our modular workflow.

The integration workflow demonstrates that we take advantage of several annotation tools to extract concepts and relations from the textual information. In this case, the cTAKES delivers respective annotations in the standoff format (http://2013.bionlp-st.org/file-formats), where the annotations are stored separately from the annotated text, and the Neji system supplies annotations in the BioC format, a verbose XML format for data exchange. Using *Ann2RDF* modular algorithms (26), all the resulting annotations can be integrated into a common and sharable interface. Concepts and relations are independently extracted from the annotation data through advanced ETL processes. Ontology mapping procedures can also be used to enrich the integrated data through configuration properties—annotation tags mappings (i.e. classified concept categories, not concept semantic identifier) and properties mappings (i.e. associations between concepts) are supported. For instance, if an entity term is recognized as a *Gene_expression* tag, the system allows this linkage to be enriched by adding new mappings to terms available in an adequate ontology (e.g. Gene Regulation Ontology—http://purl.bioontology.org/ontology/GRO#GeneExpression). Moreover, it is possible to configure external services to enrich the detected entities with normalization and disambiguation features.

**Figure 5.**
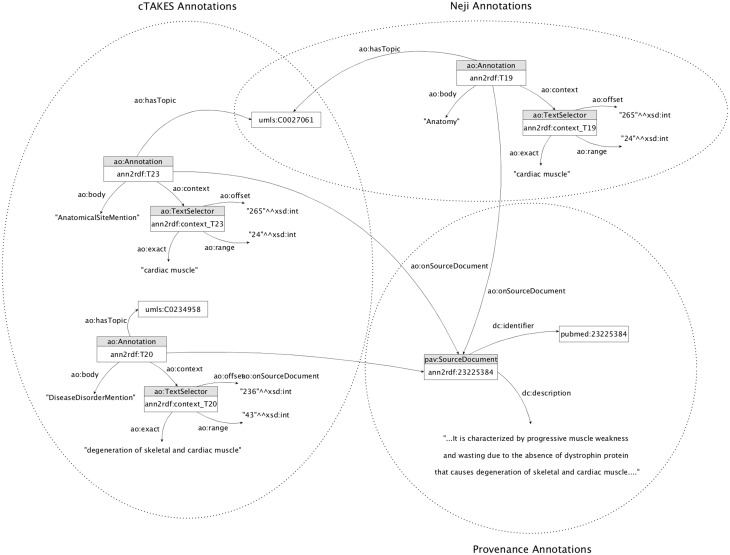
Knowledge base sample annotation model. The annotators involved share concept attributions (i.e. umls: C0027061), increasing the likelihood of being correctly identified.

These integration mechanisms are responsible for performing an adequate linkage between the information extracted by the text-mining tools and the respective adopted model. The entire workflow generated a unified knowledge base with >3.5 million triples of concepts, relations and respective provenance information (Figure 5).

Finally, the integrated information can be combined with existing and related knowledge due to its compatibility with semantic web standards and queried over SPARQL engines.

For instance, it is very straightforward to find the documents where a specific concept was identified (e.g. Skeletal muscle atrophy):

SELECT DISTINCT? source {

   *? annotation a ao: Annotation.*

   *? annotation ao: hasTopic umls: C0234958.*

   *? annotation ao: onSourceDocument? source.*

}

The knowledge base from this example can be explored through a set of semantic services available at (http://bioinformatics.ua.pt/dmd/scaleus/). Access is through a SCALEUS (35) instance, offering a public SPARQL endpoint with data federation capabilities and supporting real-time inference mechanisms. Optimized text searches over the knowledge base are also available.

## Discussion

In recent years, the number of biomedical information extraction systems has been growing steadily. The latest approaches use computational tools to help in the extraction and storage of relevant concepts, as well as their respective attributes and relationships. The product of these complex workflows provides valuable insights into the overwhelming amount of biomedical information being produced. However, interoperability issues in this domain are critical. In this manuscript, we propose an interoperable architecture to unify document curation results and enable their proper exploration through multiple interfaces geared toward bioinformatics developers and general life science researchers. This enables a unique scenario where heterogeneous results from annotation tools are harmonized and further integrated into rich semantic knowledge bases. Compared with existing techniques, our approach integrates several main features:

- The possibility to use and combine text-mined information from different and independent annotation tools.
- The adoption of a unique and effective ontology model that is currently being used by the W3C community.
- The provision of enriched information resulting from the ontological terms mapping process and the combination of text-mined results.
- Fast creation of semantic-powered knowledge bases.
- Information sharing mechanisms are simplified by using semantic web standards and adequate provenance methods.
- Finally, it enables the exploration of a multitude of semantic web technologies and services such as reasoning capabilities, Linked Data and SPARQL query endpoints.

Taking advantage of these features, we have implemented a case study regarding *DMD* disease, resulting in the integration of two text-mined solutions to analyse 2783 abstracts. The outcome is a fully-connected knowledge base of annotations allowing the exploration of complex interactions between the identified concepts. Additional semantic services combination empowers our final results, delivering enhanced information sharing and discovery methods. Ultimately, the approach developed envisages providing a modular architecture for textual information integration, normalising access and exploration. Moreover, the possibility to combine information from several annotation tools allows enhanced forthcoming quality controls, resulting in a fast strategy to identify gaps between the mined information. Using quick and optimized searches over the formalized knowledge base, information can be compared, differentiated and measured according to the user’s needs.

Finally, the general architecture of the solution allows its application in the most diverse life science scenarios. For instance, our approach was also used to convert 16 000 textual radiology reports into a knowledge base with >6.5 million triples (44). In that case, narrative reports were extracted from an SQL database and processed with just one text-mined solution. The outcome was a radiology knowledge base of clinical annotations, currently being used for medical decision support purposes.

## Conclusions

Information extraction systems have been increasingly adopted to facilitate the processing of textual information. The heterogeneity of these tasks, regarding the extraction process, generates a vast quantity of miscellaneous data, which are dependent on the systems used and, in most of the cases, are not interoperable. Despite current research efforts, advanced exploration, integration or comparison of these valuable data have been left outside the research path. We proposed a modular framework where these limitations can be overcome. Our solution resides in a fast mechanism to integrate knowledge extracted from several text-mining solutions, enabling the easy creation of semantic-powered databases. The ability to process annotations from several and miscellaneous annotation formats benefits accessibility methods, allowing the integration of heterogeneous formats into a common and interoperable model. This is the major outcome of the implemented solution. To validate our system, we extracted annotations from the scientific literature, using two different text-mining solutions, leading to the creation of a unified semantic knowledge base. Data exploration methods can be easily applied through several services, making the analysis of extracted knowledge feasible. The created repository follows Linked Data standards, facilitating the application of modern knowledge discovery mechanisms (e.g. reasoning).

## Funding

The research leading to these results has received funding from the European Community’s Seventh Framework Programme (FP7/2007-2013) under Grant Agreement No. 305444—the RD-Connect project. Pedro Sernadela is funded by Fundação para a Ciência e Tecnologia (FCT) under the Grant Agreement SFRH/BD/52484/2014.

*Conflict of interest*. None declared. 

## References

[1] Rebholz-Schuhmann D., Oellrich A., Hoehndorf R. (2012) Text-mining solutions for biomedical research: enabling integrative biology. Nat. Rev. Genet., 13, 829–839. 23150036 10.1038/nrg3337

[2] Khare R., Leaman R., Lu Z. (2014) Accessing Biomedical Literature in the Current Information Landscape. In: Kumar V., Tipney H. (eds). Methods in Molecular Biology (Methods and Protocols), vol 1159. Humana Press, New York. 10.1007/978-1-4939-0709-0_2 PMC4593617 24788259

[3] AlexGrover B.C., Haddow B. (2008) Assisted curation: does text mining really help? Pacific Symp. Biocomput, 13. 18229715

[4] Nadkarni P.M., Ohno-Machado L., Chapman W.W. (2011) Natural language processing: an introduction. J. Am. Med. Inform. Assoc., 18, 544–551. 21846786 10.1136/amiajnl-2011-000464 PMC3168328

[5] Campos D., Matos S., Oliveira J. (2012) Current methodologies for biomedical named entity recognition. Biol. Knowl. Discov. Handb., 839–868.

[6] Jimeno-Yepes A., Aronson A. (2010) Knowledge-based biomedical word sense disambiguation: comparison of approaches. BMC Bioinformatics, 11, 569. 21092226 10.1186/1471-2105-11-569 PMC3001745

[7] Zhu F., Patumcharoenpol P., Zhang C. (2013) Biomedical text mining and its applications in cancer research. J. Biomed. Inform, 46, 200–211. 23159498 10.1016/j.jbi.2012.10.007

[8] Bui Q.-C., Katrenko S., Sloot P.M.A. (2011) A hybrid approach to extract protein-protein interactions. Bioinformatics, 27, 259–265.http://dx.doi.org/10.1093/bioinformatics/btq620 21062765 10.1093/bioinformatics/btq620

[9] Tari L., Anwar S., Liang S. (2010) Discovering drug-drug interactions: a text-mining and reasoning approach based on properties of drug metabolism. Bioinformatics, 26, i547–i553., 20823320 10.1093/bioinformatics/btq382 PMC2935409

[10] Wiegers T.C., Davis A., Cohen K.B. (2009) Text mining and manual curation of chemical-gene-disease networks for the Comparative Toxicogenomics Database (CTD). BMC Bioinformatics, 10, 326 http://dx.doi.org/10.1186/1471-2105-10-326 19814812 10.1186/1471-2105-10-326 PMC2768719

[11] Topić P., Stenetorp S., Pyysalo G. (2012 ) BRAT: a web-based tool for NLP-assisted text annotation. *Proc. Demonstr. 13th Conf. Eur. Chapter Assoc. Comput. Linguist.*, pp. 102–107.

[12] Salgado D., Krallinger M., Depaule M. (2012) MyMiner: a web application for computer-assisted biocuration and text annotation. Bioinformatics, 28, 2285–2287.http://dx.doi.org/10.1093/bioinformatics/bts435 22789588 10.1093/bioinformatics/bts435

[13] Rak R., Rowley A., Black W., Ananiadou S. (2012) Argo: an integrative, interactive, text mining-based workbench supporting curation. Database (Oxford), 2012, bas010. 22434844 10.1093/database/bas010 PMC3308166

[14] Campos D., Lourenco J., Matos S., Oliveira J.L. (2014) Egas: a collaborative and interactive document curation platform. Database, 2014, bau048. 24923820 10.1093/database/bau048 PMC4207226

[15] Döring K., Grüning B.A., Telukunta K.K. (2016) PubMedPortable: a framework for supporting the development of text mining applications. PLoS One, 11, e0163794. 27706202 10.1371/journal.pone.0163794 PMC5051953

[16] Kirsch D., Rebholz-Schuhmann H. (2006) IeXML: towards an annotation framework for biomedical semantic types enabling interoperability of text processing modules. *SIG BioLink, ISMB*.

[17] Comeau D.C., Islamaj Dogan R., Ciccarese P. (2013) BioC: a minimalist approach to interoperability for biomedical text processing. Database (Oxford), 2013, bat064. 24048470 10.1093/database/bat064 PMC3889917

[18] Machado C.M., Rebholz-Schuhmann D., Freitas A.T., Couto F.M. (2015) The semantic web in translational medicine: current applications and future directions. Brief. Bioinform, 16, 89–103. 24197933 10.1093/bib/bbt079 PMC4293377

[19] Laurila J.B., Naderi N., Witte R. (2010) Algorithms and semantic infrastructure for mutation impact extraction and grounding. BMC Genomics, 11, S24. 21143808 10.1186/1471-2164-11-S4-S24 PMC3005927

[20] Coulet A., Garten Y., Dumontier M. (2011) Integration and publication of heterogeneous text-mined relationships on the Semantic Web. J. Biomed. Semantics, 2, S10, 10.1186/2041-1480-2-S2-S10 PMC3102890 21624156

[21] Mendes P.N., Jakob M., García-Silva A., Bizer C. (2011) DBpedia spotlight, in *Proceedings of the 7th International Conference on Semantic Systems—I-Semantics’11*, pp. 1–8.

[22] Lehmann J., Isele R., Jakob M. (2014) DBpedia—a large-scale, multilingual knowledge base extracted from Wikipedia. Semant. Web.

[23] Kim J., Wang Y. (2012) PubAnnotation: a persistent and sharable corpus and annotation repository. *Proc. 2012 Work. Biomed. Nat. Lang. Process. Assoc. Comput. Linguist.*, 202–205.

[24] Harris S., Seaborne A., Prud’hommeaux E. (2013) SPARQL 1.1 query language. *W3C Recomm.*, 21.

[25] Rak R., Batista-Navarro R.T., Carter J. (2014) Processing biological literature with customizable Web services supporting interoperable formats. Database, 2014, bau064., 25006225 10.1093/database/bau064 PMC4086403

[26] Sernadela P., Matos S., Oliveira J.L. (2015) Ann2RDF: moving annotations to semantic web. *Proceedings of the 17th International Conference on Information Integration and Web-based Applications &Services–iiWAS’15*. pp. 1–5.

[27] Weibel S. (2005) The Dublin core: a simple content description model for electronic resources. Bull. Am. Soc. Inf. Sci. Technol., 24, 9–11.

[28] Noy N.F., Shah N.H., Whetzel P.L. (2009) BioPortal: ontologies and integrated data resources at the click of a mouse. Nucleic Acids Res., 37, W170–W173. 19483092 10.1093/nar/gkp440 PMC2703982

[29] Nunes T., Campos D., Matos S., Oliveira J.L. (2013) BeCAS: biomedical concept recognition services and visualization. Bioinformatics, 29, 1915–1916.http://dx.doi.org/10.1093/bioinformatics/btt317 23736528 10.1093/bioinformatics/btt317

[30] Ciccarese P., Ocana M., Garcia Castro L.J. (2011) An open annotation ontology for science on web 3.0. J. Biomed. Semantics, 2, S4. 10.1186/2041-1480-2-S2-S4 PMC3102893 21624159

[31] Ding L., Michaelis J., McCusker J., McGuinness D.L. (2011) Linked provenance data: a semantic web-based approach to interoperable workflow traces. Futur. Gener. Comput. Syst., 27, 797–805.

[32] Curcin V., Miles S., Danger R. (2014) Implementing interoperable provenance in biomedical research. Futur. Gener. Comput. Syst., 34, 1–16.

[33] Kim J.-D., Ohta T., Pyysalo S., Kano Y., Tsujii J. (2009) Overview of BioNLP’09 shared task on event extraction pp. 1–9.

[34] Lopes P., Oliveira J.L. (2012) COEUS: ‘semantic web in a box’ for biomedical applications. J. Biomed. Semantics, 3, 11. 23244467 10.1186/2041-1480-3-11 PMC3554586

[35] Sernadela P., González-Castro L., Oliveira J.L. (2017) Scaleus: semantic web services integration for biomedical applications. J. Med. Syst. 41, 54. 28214993 10.1007/s10916-017-0705-8

[36] Wilkinson M.D., Vandervalk B., McCarthy L. (2011) The semantic automated discovery and integration (SADI) web service design-pattern, api and reference implementation,”. J. Biomed. Semantics, 2, 8. 22024447 10.1186/2041-1480-2-8 PMC3212890

[37] Belleau F., Nolin M.-A., Tourigny N. (2008) Bio2RDF: towards a mashup to build bioinformatics knowledge systems. J. Biomed. Inform., 41, 706–716. 18472304 10.1016/j.jbi.2008.03.004

[38] Jupp S., Malone J., Bolleman J. (2014) The EBI RDF platform: linked open data for the life sciences. Bioinformatics, 30, 1338–1339.http://dx.doi.org/10.1093/bioinformatics/btt765 24413672 10.1093/bioinformatics/btt765 PMC3998127

[39] SernadelaLopes P.P., Oliveira J.L. (2016) A knowledge federation architecture for rare disease patient registries and biobanks. J. Inf. Syst. Eng. Manag., 1, 83–90.

[40] Freitas A., Curry E., Oliveira J.G., O'riain S. (2012) Querying heterogeneous datasets on the linked data web: challenges, approaches, and trends. IEEE Internet Comput., 16, 24–33.

[41] Campos D., Matos S., Oliveira J. (2013) A modular framework for biomedical concept recognition. BMC Bioinformatics, 14, 281. http://dx.doi.org/10.1186/1471-2105-14-281 24063607 10.1186/1471-2105-14-281 PMC3849280

[42] Savova G.K., Masanz J.J., Ogren P.V. (2010) Mayo clinical text analysis and knowledge extraction system (cTAKES): architecture, component evaluation and applications. J. Am. Med. Informatics Assoc. 17, 507–513.http://dx.doi.org/10.1136/jamia.2009.001560 10.1136/jamia.2009.001560 PMC2995668 20819853

[43] Bodenreider O. (2004) The unified medical language system (UMLS): integrating biomedical terminology. Nucleic Acids Res. 32, D267–D270.http://dx.doi.org/10.1093/nar/gkh061 14681409 10.1093/nar/gkh061 PMC308795

[44] Monteiro E., Sernadela P., Matos S., Costa C., Oliveira J.L. (2016) Semantic knowledge base construction from radiology reports. *Proceedings of the 9th International Joint Conference on Biomedical Engineering Systems and Technologies*, pp. 345–352.

